# Risky sexual behavior and associated factors among sexually-active unmarried young female internal migrants working in Burayu Town, Ethiopia

**DOI:** 10.1371/journal.pone.0240695

**Published:** 2020-10-21

**Authors:** Ararso Baru, Ikeola A. Adeoye, Adeyemi O. Adekunle

**Affiliations:** 1 College of Medicine and Health Sciences, Arbaminch University, Arbaminch, Ethiopia; 2 Institute of Life and Earth Science (Including Health and Agriculture), Pan African University, Ibadan, Nigeria; 3 Slum and Rural Health Initiative Network/Ethiopia, Ethiopia; 4 Department of Epidemiology and Medical Statistics, Faculty of Public Health, College of Medicine, University of Ibadan, Ibadan, Nigeria; 5 Department of Obstetrics and Gynecology, Faculty of Clinical Medicine, College of Medicine, University of Ibadan, Ibadan, Nigeria; University of Westminster, UNITED KINGDOM

## Abstract

**Background:**

Young female internal migrants are highly vulnerable to risky sexual behaviors (RSB) which may result in serious health problems such as unintended pregnancy, abortion and sexually transmitted infections including HIV. RSB includes early sexual debut (before 18 years), having multiple sexual partners, sex without a condom or inconsistent use of condom and sex under the influence of substance use. This study aimed to assess the magnitude and the factors associated with RSB among sexually-active unmarried young female internal migrants in Burayu Town, Ethiopia.

**Methods:**

A cross sectional study design was used for the study. A total of 267 respondents was recruited into the study using a simple random sampling technique. A semi-structured interviews-administered questionnaire was used to obtain information from the study participants. The collected data were cleaned, coded and entered into Epi data version 3.1 and then exported to SPSS Ver.21 for analysis. Multiple logistic regression models were used to indicate the association between dependent and independent variables.

**The results:**

About 35% of the young female internal migrants had sexual debut before the age of 18 years; 64.4% had sex without condom or inconsistently used condom; nearly one quarter of the participants had multiple sexual partners, and 29.6% had sex under the influence of substance uses. The magnitude of RSB among the study participants was (79.1%). Sexting [AOR 3.47(95%; CI;1.10–11.94)], frequent engagement in social media [AOR 10.9(95%;CI;2.31–51.89)], feeling of embarrassment to buy condom [AOR 8.28(95%; CI; 2.10–32.62)], unfavorable attitude toward using condom for steady and loving relationship [AOR 5.72(95%; CI; 1.47–22.24)] were related with RSB while self-efficacy [AOR 0.15(95%: CI; 0.04–0.57)] to use condom and perceived risks of getting pregnant [AOR 0.05(95%; CI; 0.01–0.23)] were found to be protective factors.

**Conclusion:**

The study found high levels of RSB among sexually-active unmarried young female internal migrants. This finding suggests an urgent need of intervention to promote safe sex among this group. Special attention and prompt interventions are needed to promote the use of condoms.

## Background

Ethiopia is one of the developing countries in Sub-Saharan Africa with an estimated 80 percent of the population inhabited in the rural area [1]. Notwithstanding, the recent urbanization rate in the country is rapidly increasing at about 5.4 percent per year, and by 2028 the country’s population in urban areas will reach 30 percent [2]. Internal migration (mainly rural to urban) is the main reason for the rapid increase [3].

Internal migrations in Ethiopia are due to social, economic, climatic and political factors like elsewhere in sub-Saharan Africa [4, 5]. The reasons include but not limited to factors such as drought, war, famine, political turmoil, forced migrations, education, searching for a better job opportunity and marriages, or escaping from early marriages [4, 6, 7]. Young people are a dominant group among all internal migrants in the country [6–8]. However, they are an economically disadvantaged group with little education and limited skills [6, 9].

Migration during the formative adolescent and early adult years can affect life-course transitions through several pathways and can lead young people to risky sexual behaviors [10, 11]. First, migration can alter existing sexual partnerships and provide mobile individuals with an opportunity to acquire new partners. Secondly, migrants are physically separated from their usual social norms and networks so that they may experience social isolation [12, 13]. Thirdly, they also might feel emotionally distanced from partners at home [12, 14]. Moreover, migrants have limited knowledge and access to reproductive health services [15, 16]. So, they are more likely to engage in risky sexual behaviors than non-migrants [12, 14, 17, 18]. Risky sexual behavior may result in serious health problems such as unintended pregnancy, abortion, and sexually transmitted infections (STIs) including HIV [19].

In Ethiopia, risky sexual behavior was higher among migrants than non-migrants [6, 7, 20–22]. According to the study conducted in Northwest Ethiopia, 80.4% of young female internal migrants were engaged in risky sexual practices [21]. Indeed, there is a high prevalence of multiple sexual partners, less utilization of condoms, and higher proportion of commercial sex engagement among migrants than non-migrants in the country [6].

Most studies conducted on risky sexual behaviors in Ethiopia were mainly focused on key populations such as female sex workers, secondary school students, university students, adolescents, youths, and substance users [6, 20, 21, 23–25]. Nevertheless, the study that focused on the sexual behavior of internal migrants is very limited. As a result, very little is known about the relationship between migration and risky sexual behavior among young female internal migrants in Ethiopia. Therefore, this study examined risky sexual behavior and associated factors among sexually-active unmarried young female internal migrants working in Burayu town, Ethiopia.

## Objectives

The specific objectives were:

- To determine the magnitude of risky sexual behaviors among sexually-active unmarried young female internal migrants working in Burayu town, Ethiopia
- To identify the factors associated with risky sexual behaviors among sexually-active unmarried young female internal migrants working in Burayu town, Ethiopia

## Methods

### Study design

Cross-sectional study design was conducted to assess the magnitude and factors associated with risky sexual behaviors among sexually active unmarried young female internal migrants.

### Study setting and period

The study was conducted in Burayu town, Ethiopia. The town is located 10 km to the West of Addis Ababa, the capital city of Ethiopia. The town has six kebele (the smallest administrative unit in Ethiopia). The town’s strategic locations (near Addis Ababa city) and the availability of infrastructures attracted domestic and foreign investors to the town. As a result, a large number of young people mostly from rural and semi-urban areas move to the town to find better work opportunities. This study included three of the kebele located in Burayu town namely Lakku Katta, Burayyu Katta, and Galarza Nonno. The study was done from April to June 2019 among participants recruited from factories, restaurants, hotels, cafeterias, bars, and beauty salons located in the selected kebele.

### Study participants

The source population was all sexually active unmarried young female (aged 15–24 years) internal migrants working in Burayu town, Oromia, Ethiopia. Eligibility criteria included being female, being in the age group of 15–24 years, who had the experience of penetrative sexual intercourse over the last six months, unmarried, internal migrants, employed in one of the recruitment settings, and have consented to participate in the study. Thus, this study included participants selected from three of the kebele located in Burayu town namely Lakku Katta, Burayyu Katta and Galarza Nonno based on eligibility criteria.

### Sample size determination

The sample size (n) was determined based on a single population proportion formula with the following assumptions. The prevalence of risky sexual behavior among young female internal migrants was 80.4%, which was taken from a study conducted in Tiss Abay town, Ethiopia [21]. The level of confidence (α) was set at 0.05 (Z (1-α) = 1.96) and the margin of error was taken as 0.05. Accordingly, the calculated sample size with 10% consideration for non-response rate was 267.

$$n=\frac{{\left[Z(\alpha )\right]}^{2}xP(q)}{d^{2}}$$

Where; n = Minimum sample size for a statistically significant survey

Z = Standard normal deviate at 95% confidence interval two-tailed test is; = 1.96

P = Prevalence of risky sexual behavior among young female internal migrants was considered as 80.4%

q = 1-p, d = margin of error taken as 5% = 0.05

### Sampling procedures

Prior to recruitment, informational leaflets about the survey were distributed to the staff in each recruitment site and 783 eligible individuals showed interest for participation in to the study. Then, sample frame was developed for the interested young female and 267 participants were recruited to the study based on the calculated sample size using simple random sampling technique. All the eligible women gave informed consent to take part in the study. To select the study subjects, sampling frames were developed from leaflets of individuals who were eligible for the study. Therefore, study subjects were selected by simple random sampling technique.

### Variables

#### Dependent variable

The dependent variable in this study was risky sexual behavior which included: multiple sexual partners; sex without condoms or inconsistent condoms use; initiation of sex before the age of 18 years; sexual intercourse under the influence of substances

#### Independent variable

Independent variable of the study included: socio-economic and demographic variables such as age, educational status, monthly income, religiosity, parental youth communication on sexuality, migration types (rural-urban, urban-urban) and duration time since the migration to Burayu town; motivational & behavioral factors such as sexual behaviors with men (the type of sexual relationship, sex for money exchange, transactional sex, intergenerational sex and among others), substance use (alcohol, khat, cigarettes, among others), social media use (Facebook, WhatsApp, IMO, Instagram etc), exposure to pornography, types of the sexual partner (male clients, boyfriends or lovers and casual partners) and peer pressures; psychological & cognitive factors which included variable such as perceived self-efficacy to avoid sex without condoms, risk perception (STIs and pregnancy) and attitude toward condom use.

### Data collection techniques and instrument

An interviewer-administered structured questionnaire was used to collect information from each participant. The English version of the questionnaire was adopted from a WHO standard questionnaire on sexual and reproductive behaviors of young people with modifications to fit the objectives of the study. The questionnaire had both open, and close-ended questions to address all questions about the study. The key factors that were associated with young female internal migrant’s risky sexual behaviors were organized and classified as socio-economic and demographic characteristics, behavioral and motivational characteristics, and cognitive and psychological aspects.

### Measurement

Risky Sexual behavior measurement: for the purposes of this study, RSB was considered as initiating sexual debut before the age of 18, not using or inconsistent condom use, sex under the influence of substances, and having multiple sexual partners. Other studies also recognized these as a measure of risky sexual behavior [20, 26]. The participants were asked all of the questions created to measure risky sexual behavior. These variables are defined as (a) Subjects had more than one sex partners at a time over the last six months in a period after migration, (b) The respondent initiated the first sexual intercourse before age of 18 years in the period after migration, or currently less than 18 years old and experienced penetrative sexual intercourse over the last six months in a period after migration but initiated sexual intercourse before migration, (c) Subjects had sexual intercourse without a condom or inconsistent use of condom during the last 6 months preceding the date of the survey and in the period after migration, (d) Subjects had sexual intercourse under influence of substance use (like alcohol, khat, and shisha) during the last 6 months preceding the date of the survey and in the period after migration. An individual was considered as engaging in risky sexual practice if she experienced at least one or more of the above behaviors [20, 26].

Perceived risks of contracting HIV/STIs were measured by the questions that asked "Were you ever concerned that you might contract AIDS or another sexually transmitted disease from your sexual partner?” This dichotomous measure was coded 1 = yes if they had ever used a condom, 0 if otherwise.

Knowledge about male condom was measured based on the questions about condom knowledge, which were: **“**having heard about the male condom; have you ever seen a male condom in demonstration?; do you know that condom prevents unplanned pregnancy?; do you know that condom prevents HIV/AIDS and STIs?” These dichotomous measures were coded 1 = yes and 0 if otherwise. In addition, an individual was asked the following questions on the care needed for correct use of the male condom. These questions were: “I always check the expiry date of condom; I don’t open the package with the mouth/teeth; Condom should be put on before sexual intercourse; I check the integrity of the package/condom before use; I hold the tip of the condom so that no air gets in; A male condom should be put on only if the penis is fully erect; a male condom can be used more than once” These variables were coded 1 = agree and 0 if otherwise. An individual who was agreed on the following questions were categorized as they had adequate knowledge about condoms. (a) having heard about the male condom (b) had ever seen a male condom in demonstration (c) had knowledge that they prevent HIV/STD (d) had knowledge that they prevent unplanned pregnancy and being able to mention at least three forms of care needed for the correct use of the male condom. categorized as they had adequate knowledge about condoms.

### Data quality assurance

The qualities of data were assured through careful design, translation, and retranslation of the study tool language from English version to local languages (Amharic and Afan Oromo) and vice versa. The questionnaire was pre-tested before data collections and possible corrections were made. Besides, two days training was given for the data collectors and supervisors. The confidential face-to-face survey interviews were conducted by trained female data collectors of similar age to the participants due to the sensitive nature of the queries. In addition, the pilot test was done but the results were not included in the actual study. Based on the feedback from the pilot study, immediate corrective measures were taken. Furthermore, continuous and close supervision of the data collecting procedures, proper categorization, and coding of the data was done. The Principal Investigators and the supervisors checked the completeness and consistency of data on a daily basis.

### Data entry, processing and analysis

Data were checked for completeness and inconsistencies. Then, they were cleaned, coded, and entered into EPI data for validation. Lastly, it was exported to Statistical Package for Social Science (SPSS) version 21 for analysis.

Descriptive statistics were used to summarize the data while the table and diagrams were used to present information. Binary logistic regression was used to observe associations between dependent and independent variables. Purposive selections of the variable with a p-value of < 0.25 on bivariate analysis were considered for binary logistic regression to control the effect of other confounders. Then, a significant level was set at p<0.05.

### Ethics approval and consent to participate

Ethical clearance was obtained from the University of Ibadan /University College Hospital (UI/UCH) Ethical Review Board (Certificate of Ethical Clearance No: 18/0550). Similarly, clearance was obtained from the Oromia Regional Health Bureau (ORHB). The ORHB directed the Burayu town’s health Bureau and respective institutions to allow the study to be conducted.

The purpose of the study was explained and verbal consent was obtained from each study participant prior to the interviews. Verbal assent was also secured from the parent or guardian of each dependent participants. The respondents were informed that they had the right to be involved or refuse to participate in the study. In addition, the respondent had the right to withdraw from the study at any time during the interview. The participants were assured that the data would be handled exclusively by the investigators and no one would be able to recognize them in the report. The confidentiality of the information obtained from each participant was maintained.

## Results

### Socio-demographic characteristics of the participants

The majority of (153{57.3%}) the participants were in the 15–19 years’ age (with a mean = of 18.85 years), (SD = 2.337). With regard to religious affiliation, the majority (123{46.1%}) of the participants were protestants, and 98(36.7%) were Ethiopian Christian orthodox religious followers. In addition, nearly half (124{46.4%}) of the study participants attended religious service more than once per week (Table 1).

**Table 1:** Percentage distribution of socio-demographic characteristics of sexually active unmarried young female internal migrants in Burayu Town, Ethiopia.

Variables	Frequency (N = 267)	Percentage (%)
**Age of respondent**		
(*Mean = 18*.*85 years*, *S*.*D = 2*.*337*,*Variance = 5*.*464)*		
15–19 years	153	57.3
20–24 years	114	42.7
**Religious affiliation**		
Protestant	123	46.1
Ethiopian Orthodox	98	36.7
Muslim	29	10.9
Others	17	6.4
**Frequency of attending religious service**		
More than once a week	124	46.4
More than once a month	101	37.8
Never attend	42	15.7
**Educational Status**		
Primary	46	17.2
Secondary	116	43.4
Tertiary	105	39.3
**Currently studying**		
Yes	97	36.3
No	170	63.7
**Place of residence before migration**		
Urban	126	47.2
Rural	141	52.8
Length of time stayed in Burayu		
<2 years	138	51.7
2–5 years	74	27.7
>5 years	55	20.6
Live with at least one member of their family		
Yes	74	27.7
No	193	72.3
Monthly income in Ethiopian Birr*		
<1000	74	27.7
1000–2000	153	57.3
>2000	40	15.0

### Behavioral characteristics of study participants

This study revealed that more than one-fourth (73{27.3%}) of the study participants went to the clubs over the last six months while one-fifth (53{19.9%}) of them went to the cinema or movie houses. Ninety-two (34.5%) study participants watched pornography at least once over the last six months. Nearly three-fourth of (195{73.0%}) the study participants used social media, and the majority of (107{54.9%}) them used it many times daily. In addition, 94(48.4%) respondents who were active on the social media engaged in sexting with men over the last six months [Table 2].

**Table 2:** Percentage distribution of behavioral characteristics of sexually active unmarried young female internal migrants in Burayu Town, Ethiopia.

Variables	Frequency (N = 267)	Percentage (%)
**Went to clubs or parties over the last 6 months**		
Yes	73	27.3
No	194	72.7
**Went to cinema house over the last 6 months**		
Yes	53	19.9
No	214	80.1
**Currently use alcohol**		
Yes	136	50.9
No	131	49.1
**Currently chew Khat**		
Yes	47	17.6
No	220	82.4
**Currently smoked cigarettes/weeds**		
Yes	63	23.6
No	204	76.4
**Watched pornography**		
Yes	92	34.5
No	175	65.5
**Use social media**		
Yes	195	73.0
No	72	27.0
Frequency of social media use*		
Many times, a day	107	54.9
Several times a week	43	22.1
Once a while	45	23.1
Participated in sexting over social media with men*		
Yes	94	48.2
No	101	51.8

‘*’ Indicates N = 195

With regard to substance use, more than half (136{50.9%}) of the participants used alcohol, 47(17.6%) chewed khat while 63{23.6%) respondents smoked cigarettes or weeds [Table 2].

## Discussion

**Fig 1:**
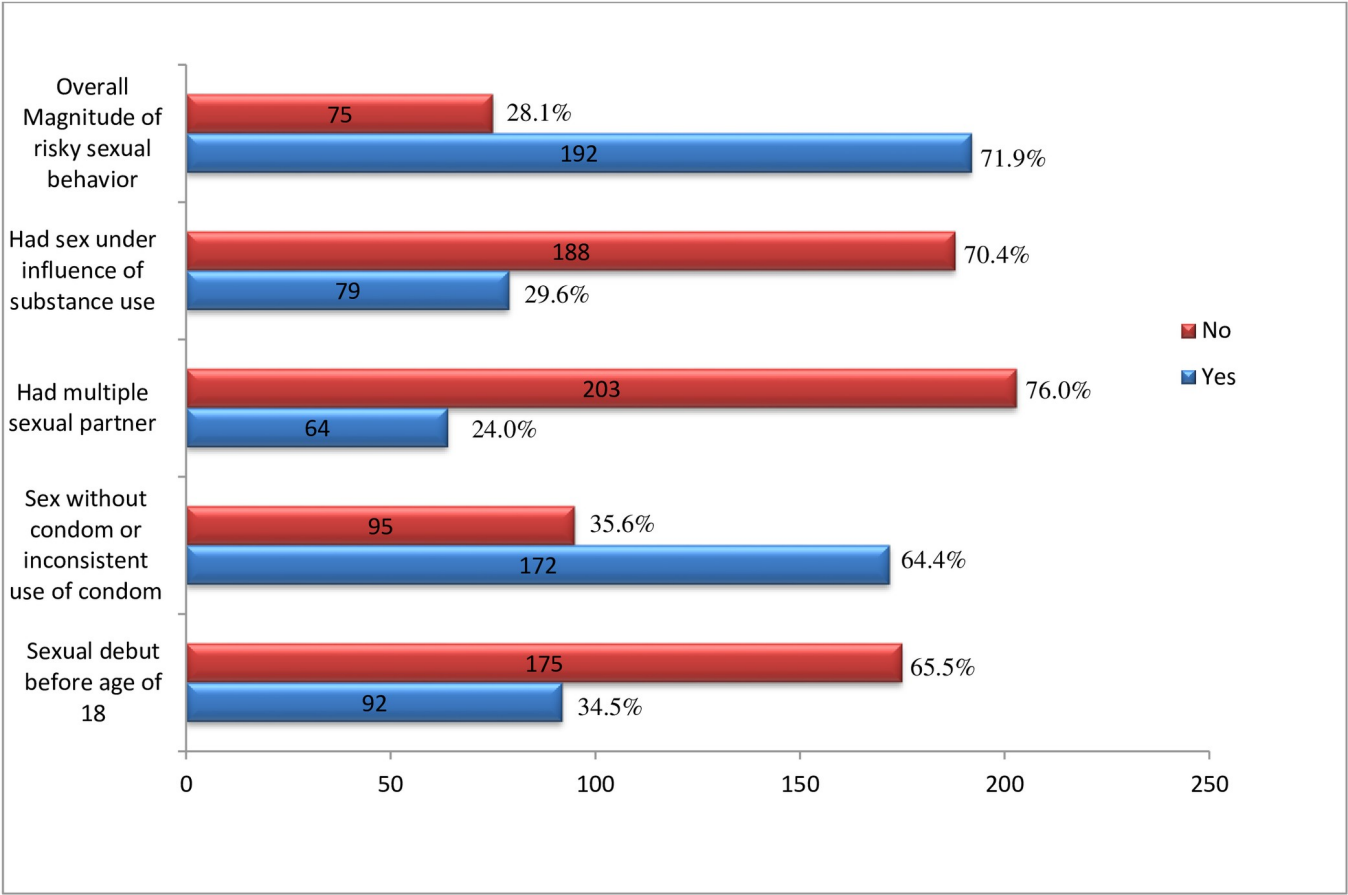
Magnitude of risky sexual behavior among sexually-active unmarried young female internal migrants in Burayu Town, Ethiopia.

In this study, 34.5% of the study participants had sexual debut before the ages of 18 years [Fig 1]. This finding is higher than found among a comparable sample of young migrant women in China [17] but lower than among young working in hair salons in Nigeria [27]. The high prevalence of early sexual debut in this study might be explained by poverty, unemployment, lack of comprehensive knowledge on the impact of early sexual initiation, being less connected with parents, being from the rural community (in this study the most of the internal migrants were rural-urban migrants), alcohol drinking and exposure to pornographic materials [23, 28–30].

Nearly one-fourth (23.97%) of the study participants had multiple sexual partners during the last six months [Fig 1]. Similar findings have been reported among female internal migrant workers in China [31]. On the contrary, the present finding was slightly lower than a comparable study conducted among university students in Ethiopia, which reported (30.14%) prevalence of multiple sexual partners [32]. It was evidenced that males were more likely to have multiple sexual partners compared to women in Ethiopia [33]. In fact, the study conducted among university students in Ethiopia has included both male and female respondents while the present study included female respondents only. Therefore, the disparity in the prevalence of multiple sexual partners could be attributable to this.

This study found that 64.4% of the respondents had sex without a condom or inconsistently used condoms in their most recent sexual intercourse during the last six months [Fig 1]. This was supported by a study conducted among unmarried female migrants in Shanghai which reported, 66.8% prevalence [34] and relatively lower compared to another study conducted among female internal migrants workers in China which reported 84% [31]. However, this doesn’t necessarily mean the finding of the present study is acceptable compared to the higher prevalence reported in China. The high proportion of sex without a condom or inconsistent use of a condom in the present study could be explained by the existence of a considerable proportion of transactional sexes in this study which was definitely associated with unprotected sex as reported by earlier studies conducted elsewhere [18, 21, 35, 36]. It could also be attributed to an unfavorable attitude toward using a condom or the social norms in which the youth live and grow. For instance, using a condom is considered as distrusting sexual partners by some people in Ethiopia and a significant proportion of internal migrants in the country reported that either they or their sexual partner do not like condom [21].

This study found that about 72% of the respondents reported at least one form of risky sexual behavior, which was slightly lower than the finding of a similar study conducted in Ethiopia (80.4%) [21]. This similarity reflects the considerable burden of RSB among internal migrants within the country. The slight disparity could be attributed to the difference in the composition of the study subject, viz., the present study was conducted among all types of internal migrants while the comparable study was done mainly among rural to urban migrants. In addition, the previous study was conducted in a semi-urban setting compared to this study. It has been reported that the migrants in small urban areas acquire reproductive health knowledge sooner than migrants in the metropolitan area [37].

More than one-third (34.5%) of the study participants watched pornography at least once over the last six months [Table 1]. The finding of this study was higher than that of a comparable study conducted among preparatory students in Southwest Ethiopia [25]. The variation in the findings could be attributed to the fact that the study from Southwest Ethiopia included both migrants and non-migrants while the present study included only internal migrants. The later has less control from family so that they could engage themselves in activities that deviate from their culture [38].

This study found that one-fourth of (25.8%) the study participants were raped by their sexual partners or strangers over the last six months preceding the data collections period [Table 3]. The studies conducted in Northwest Ethiopia among street women [39] and that held in Southern Ethiopia among university students [40] found similar results even though they reported lifetime and one-year prevalence of rape, respectively.

**Table 3:** Percentage distribution of sexuality and reproductive health characteristics of sexually-active unmarried young female internal migrants in Burayu Town, Ethiopia.

Variables	Frequency (N = 267)	Percentage (%)
**Marital status of the most recent sexual partner**		
Single	199	74.5
Married	28	10.5
Divorced	25	9.4
Separated	15	5.6
**Self-description of the relationship with partner**		
Casual	59	22.1
A serious relationship but with no intention of marriage	67	25.1
Important and might lead to marriage	86	32.2
Engaged to be married	55	20.6
**Had sex without their willingness (rape)**		
Yes	69	25.8
No	198	74.2
Discussed about SRH with ≥1 family member		
Yes	52	19.5
No	215	80.5
**Received SRH information at current destination**		
Yes	121	45.3
No	146	54.7
**Ever received SRH information at school**		
Yes	181	67.8
No	86	32.2
**Discussed about FP and STIs with their partner**		
Yes	166	62.2
No	101	37.8
**Experienced pressure for sexual intercourse**		
Yes	135	50.6
No	132	49.4
**Had planned first sex at current destination**		
Yes	68	25.5
No	199	74.5
**Had intergenerational sexual intercourse**		
Yes	82	30.7
No	185	69.3
**Gave sex after food invitation or other gifts**		
Yes	79	29.6
No	188	70.4
**Gave sex in exchange for money**		
Yes	47	17.6
No	220	82.4

Abbreviations: FP- Family planning; SRH -Sexuality and reproductive health; STIs- Sexually transmitted infections

Regarding the discussion and communication with family about SRH, only one-fifth (19.5%) of the study participants ever discussed about SRH with at least one of their family members [Table 3]. Despite the fact that the study population was different, an earlier study conducted among internal migrant children in Ethiopia reported that 21% of the migrant parents talked to them about HIV and only 11% reported discussions on sex [8].

Consequently, this study found out that about 58% of the respondents expressed the perceived risks of contracting HIV [Table 4], which was far lower than the study conducted among construction laborers in China which reported 71% [41]. This could be ascribed to a difference in the age composition of the two studies; the present study used only young people while the study from China was conducted among all age groups. In fact, older people might have higher risk perceptions than the younger age groups.

**Table 4:** Risk perception, knowledge and attitude toward condom use among sexually-active unmarried young female internal migrants in Burayu Town, Ethiopia.

Variables	Frequency(N = 267)	Percentage (%)
**Knowledge about condom**		
Adequate	101	37.8
Inadequate	166	62.2
**I feel embarrassed to buy condom**		
Agree	161	60.3
Disagree	106	39.7
**Condoms are suitable for steady and loving relationships**		
Agree	130	48.7
Disagree	137	51.3
**Condoms reduce sexual pleasure**		
Agree	129	48.3
Disagree	138	51.7
**Condoms are suitable for casual relationships**		
Agree	222	83.1
Disagree	45	16.9
**Condoms should be used in premarital sex**		
Agree	188	70.4
Disagree	79	29.6
**Using condom is distrusting your partner**		
Agree	131	49.1
Disagree	136	50.9
**A girl can suggest the use of condom**		
Agree	180	67.4
Disagree	87	32.6
**I have self-efficacy toward condom use**		
Agree	86	32.2
Disagree	181	67.8
**Perceived risks of contracting HIV/STIs**		
Yes	156	58.4
No	111	41.6
**Perceived risks of getting pregnant**		
Yes	121	45.3
No	146	54.7

A significant association was observed between the age of participants and RSB [Table 5]. It was observed that the internal migrants in the age group of 15–19 were at about two times an increased risk of risky sexual behavior compared with age group of 20–24 on crude analysis. However, after adjusting for potential covariates, the age of the study participants was no more statistically significant [Table 5]. Similarly, comparable studies were realized statistically significant association between age and RSB, in which younger age was found to be a predictor of RSB [41, 42].

**Table 5:** Factors associated with risky sexual behavior among sexually-active unmarried young female internal migrants in Burayu Town, Ethiopia.

Variables	Sexual Behavior		COR 95% CI	AOR 95% CI
	Risky	Not risky		
**Age of respondents**				
15–19 years	119	34	1.97(1.15–3.37)	1.47(0.41–5.37)
20–24 years	73	41	Ref.	Ref.
**Live with ≥1 member of their family**				
Yes	46	28	Ref.	Ref.
No	146	47	1.89(1.07–3.35)	3.69(0.79–17.06)
**Went to clubs over the last six months**				
Yes	61	12	2.44(1.23–4.86)	1.1(0.16–6.08)
No	131	63	Ref.	Ref.
**Went to cinema over the past six months**				
Yes	45	8	2.56(1.15–5.74)	3.57(0.49–26.05)
No	147	67	Ref.	Ref.
**Currently uses alcohol**				
Yes	112	24	2.98(1.69–5.23)	2.23(0.48–10.33)
No	80	51	Ref.	Ref.
**Watched Pornography over the last six months**				
Yes	75	14	2.80(1.46–5.37)	2.39(0.49–11.73)
No	113	59	Ref.	Ref.
**Frequency of using social media**				
Many times, a day	45	4	9.0(2.77–29.28)	10.9(2.31–51.89)**
Several times a week	75	26	2.31(1.10–4.83)	3.39(0.58–19.60)
Once a while	25	20	Ref.	Ref.
**Had sexting with sexual partner**				
Yes	79	15	2.67(1.34–5.32)	3.47(1.10–11.94) *
No	67	34	Ref.	Ref.
**Had involuntary sex**				
Yes	62	7	4.63(2.01–10.68)	2.61(0.41–16.45)
No	130	68	Ref.	Ref.
**Ever received SRH information at school**				
Yes	123	58	0.52(0.28–0.97)	0.34(0.07–1.79)
No	69	17	Ref.	Ref.
**Had planned the first sex after migration**				
Yes	40	28	Ref.	Ref.
No	152	47	2.26(1.26–4.06)	3.56(0.75–16.96)
**Gave sex after food invitation or other gifts**				
Yes	60	9	3.36(1.57–7.19)	15.62(0.91–267.96)
No	131	66	Ref.	Ref.
**Gave sex in exchange for money**				
Yes	42	5	3.92(1.49–10.34)	9.64(0.53–175.02)
No	150	70	Ref.	Ref.
**Able to refuse sex without condom**				
Yes	56	60	0.10(0.05–0.20)	0.15(0.04–0.57)**
No	136	15	Ref.	Ref.
**Perceived risks of contracting HIV/STIs**				
Yes	99	57	0.34(0.18–0.61)	0.37(0.10–1.43)
No	93	18	Ref.	Ref.
**Perceived risks of getting pregnant**				
Yes	67	54	0.21(0.12–0.37)	0.05(0.01–0.23)***
No	125	21	Ref.	Ref.
**Knowledge about condom**				
Adequate	61	40	0.41(0.24–0.70)	0.48(0.13–1.77)
Inadequate	131	35	Ref.	Ref.
**I feel embarrassed to buy condom**				
Agree	136	25	4.86(2.74–8.61)	8.28(2.10–32.62)**
Disagree	56	50	Ref.	Ref.
I use condom in steady and loving relationships				
Agree	75	55	Ref.	Ref.
Disagree	117	20	4.29(2.38–7.73)	5.72(1.47–22.24)*

*p<0.05

**p<0.01

*** p<0.001

Previous studies have documented enhanced vulnerability to risky sexual behavior when individuals use alcohol because of the fact that the nature of alcohol in decreasing attention to safe sexual practices, altering rational decision-making, and increasing risk-taking behaviors [43, 44]. The presents study findings also provided supportive evidence. Indeed, the young female migrants who used alcohol before sexual intercourse were about three times more likely practiced RSB than their counterparts [Table 5]. However, the association was only significant on crude analysis.

Recent technology is facilitating the tendency to have multiple sexual partners and practical sexual behavior as shown in earlier studies [45, 46]. Similarly, our study revealed that the young female who uses social media many times daily were about eleven times at increased risk of practicing RSB than the respondents who use social media occasionally [Table 5]. In agreement with this finding, a study conducted in Nigeria showed that spending much time with social media increases the risk of vulnerability to RSB among young people [47]. The finding of this study also identified that the respondents who shared sexually explicit texts, images, or/and videos with their sexual partners over social media were about 3.5 times more likely to experience RSB than those never sexted [Table 5]. Consistent with this study finding, sexting of any kind was associated with higher rates of engaging in risky sexual behaviors among adolescents in the United States [48]. On the contrary, another study from the United States showed that sexting was not associated with RSB among teenagers [45]. Further studies are recommended for a better understanding of the association between sexting and risky sexual behavior among this group.

It was clear that rape victims were vulnerable to RSB in earlier studies [39, 40]. In our study, we also found that the respondents had forced sex over the last six months preceding the field data collection were about five times more likely to have RSB than their counterparts on crude analysis [Table 5]. Even though our finding was not statistically significant on adjusted analysis, it has still public health relevance after adjusting for confounders. Indeed, female migrants who had forced sex were at 2.6 times increased risk of practicing RSB than those who had volunteered sexual intercourse [Table 5].

Young women who had received sexuality education through schools, parents, or other family members were less likely to engage in RSB [49, 50]. Our findings also supported this evidence. Accordingly, a young female migrant who received school-based sexuality and reproductive health information was 48% less likely engaged in RSB than those who never received school-based sexuality education on crude analysis. This association was no more established after adjusting for confounding variables but still have public health significance [Table 5].

The unadjusted analysis of the present study implies that the study participants who gave sex in exchange for money were nearly four times more likely to have risky sexual behavior than their counterparts. In agreement with this study, transactional sex was associated with risky sexual behavior according to the research conducted in Northwest Ethiopia [21].

Evidence suggested that the women who reported hunger were more likely to engage in transactional sex that could expose them to unprotected sexual intercourse [18, 36]. In agreement with aforementioned studies, the woman who received food invitation were over three times at increased risk of practicing RSB than their counterparts on the crude analysis of our study. Despite the fact that this finding was not statistically significant on multivariate analysis, its public health significance was more noted on the adjusted analysis. Hence, the woman who gave sex after food invitation or received the gift was over fifteen times at increased risk of unprotected sexual intercourse than the reference group [Table 5]. A possible explanation for this result could be that migrants are economically disadvantaged so that they might have been participated in risky behavior to support their daily life.

The study participants who reported a feeling of embarrassment to buy condoms were about eight times more likely to have RSB than the reference group [Table 5]. In agreement with this finding, it has been reported that embarrassment to buy condoms was a key predictor for RSB among young people [50, 51]. Moreover, the respondents who had a negative attitude about the use of condom for steady and loving relationships were about six times more likely to have RSB than those who had a positive attitude about the importance of condoms for the same purpose after adjusting for confounders, [AOR 5.72(95%; CI; 1.47–22.24)] [Table 5]. It has been reported that young people are less likely to perceive themselves at risk of contracting HIV/AIDS or unwanted pregnancy when they believe they are practicing true love with their partner [52].

The women who reported the ability to refuse sex without condoms were 85% less likely to have RSB than their counterparts in this study [Table 5]. Our finding agrees with the result of research conducted in Cameroon [53]. Therefore, it has significant implications for policy makers if they give attention to strategies that can promote self-efficacy toward the refusal of sex without condoms.

The perceived risks of getting pregnant independently predict risky sexual behavior in the present study. Accordingly, the women who reported the perceived risk of getting pregnant were 95% less likely to practice risky sexual behavior than their counterparts after adjusting for potential confounders [Table 5].

### Limitation of the study

This study results should be interpreted while considering several limitations. First, we cannot draw causal conclusions owing to the cross-sectional design of the study. Secondly, given that the sample population was limited to one city, the investigator could not assert that it is representative of all sexually-active unmarried female migrant workers in Ethiopia. Thirdly, this study was limited in its reliance on self-reported data because it is a sensitive topic; however, we tried our best to get unbiased responses. Despite these limitations, this study identified a range of major reproductive health issues related to young female internal migrants that affect their sexual behavior which was probably underexplored in Ethiopia by earlier studies.

### Generalizability

The potential generalizability of the evidence generated by this study to other settings should be considered in view of the study setting, context, methods and limitations described in this study.

## Conclusion

A considerable proportion of respondents in this study practice RSB, which could endanger their future life. Some variables were found to have been determined RSB in this study. These included sexting, frequent engagement in social media, feeling of embarrassment to buy condoms, unfavorable attitudes towards using a condom for a steady and loving relationship. Even though the association was not established after adjustment, age of respondent, family composition, the frequency at clubs or parties, a cinema house, watching pornography, alcohol use, rape, planned first sex after migration, and transactional sex was also associated with risky sexual behavior. On the other hand, perceived self-efficacy to use a condom and perceived risks of getting pregnancy, adequate knowledge of condom, school-based SRH education, parental child communication on SRH, and perceived risks of contracting HIV/STIs were found to be protective of risky sexual behavior among respondents. This finding suggests an urgent need for intervention to promote safe sex among sexually-active young female internal migrants. Special attention and prompt interventions are needed to promote the use of a condom. Such interventions should include targeting the prevailing misconceptions, unfavorable attitudes, and other dynamics related to not using a condom or inconsistent condom use which was prominent in this study. Strategies to channel young female internal migrants into productive and safe livelihoods should be emphasized to reduce rape, transactional sex, and sex work.

## Supporting information

S1 Questionnaire (DOCX)

S1 Data (SAV)

## Abbreviation

AB: Ararso Baru
AIDS: Acquired Immuno-deficiency Syndrome
AOA: Adeyemi O. Adekunle
AOR: Adjusted odds ratio
COR: Crude odds ratio
FGD: Focus Group Discussion
HIV: Human Immuno-deficiency Virus
IAA: Ikeola A. Adeoye
RSB: Risky Sexual Behaviour
SRH: Sexual and Reproductive Health
SPSS: Statistical Package for Social Sciences
STIs: Sexually Transmitted Infections
USAID: United States Agency for International Development
UNFPA: United Nations Fund for Population Activities
PAULESI: Pan African University Life And Earth Sciences Institute
