# Supplementary material for: Risky sexual behavior and associated factors among sexually-active unmarried young female internal migrants working in Burayu Town, Ethiopia
Source: PLoS One. 2020 Oct 21;15(10):e0240695. doi: 10.1371/journal.pone.0240695 (PMC7577498; doi:10.1371/journal.pone.0240695)
Supplement: S1 Questionnaire — (DOCX) [file pone.0240695.s001.docx]

| - 1. Were you born in Burayu town? | Yes 1 No 2 |  |
| --- | --- | --- |
| - 1. If not, where have you been living before migrating to Burayu town? |  |  |
| - 1. How you explain the status of the place you grew up before migrating to Burayyu town? | Urban 1  Rural 2 |  |
| - 1. For how long you stayed in Burayu town? |  |  |
| - 1. How old were you at your last birthday? | Years old |  |
| - 1. What is the highest level of schooling you completed?   (CIRCLE HIGHEST SCHOOL LEVEL) | Primary 01  Secondary 02  Preparatory 03  Technical (DIPLOMA) 04  University 05 |  |
| - 1. Are you currently attending regular school, college or university? Full-time or part-time? | Yes, full-time 1  Yes, part-time 1  No 2 |  |
| - 1. How old were you when you started working for pay? | Age in years |  |
| - 1. About how many hours a week do you work? | Hours |  |
| - 1. How much do (did) you earn in a month? | Monthly wage Enter Unit………. |  |
| - 1. What is your religion? | Orthodox 01  Muslim 02  Protestant 03  Other………………………………04  (SPECIFY) | 1.14 |
| - 1. How often do you usually attend religious services? | Every day 1  At least once a week 2  At least once a month 3  At least one a year 4  Less than once a year 5  Never 6 |  |
| - 1. How important is religion in your life? | Very important 1  Important 2  Not important 3 |  |
| - 1. Now I have some questions about your family. Is your father alive? | Yes 1  No 2 | 1.18 |
| - 1. Does he live in the same household as you? | Yes 1  No 2 |  |
| - 1. Do you find it difficult or easy to talk with your father about things that are important to you? | Very easy 1  Easy 2  Average 3  Difficult 4  Very difficult 5 |  |
| - 1. Have you ever discussed sex-related matters with your father? If YES Often or occasionally? | Often 1  Occasionally 2  Never 3 |  |
| - 1. Is your mother alive? | Yes 1  No 2 | 1.22 |
| - 1. Does she live in the same household as you? | Yes 1  No 2 |  |
| - 1. Do you find it difficult or easy to talk with your mother about things that are important to you? | Very easy 1  Easy 2  Average 3  Difficult 4  Very difficult 5  Do not see her 6 |  |
| - 1. Have you ever discussed sex-related matters with your mother? If YES Often or occasionally? | Often 1  Occasionally 2  Never 3 |  |
| - 1. Do you have any older brothers? | Yes 1  No 2 | 1.24 |
| - 1. Do any live in the same house? | Yes 1  No 2 |  |
| - 1. Do you have any older sisters? | Yes 1  No 2 | 1.26 |
| - 1. Do you live in the same house with your sister? | Yes 1  No 2 |  |
| - 1. And now I have some questions about your social activities. Do you ever go to clubs or parties where young people dance? IF YES. How many times in the last month? | Number of times Never |  |
| - 1. Do you ever go to the movies or cinema house? IF YES. How many times in the last month? | Number of times Never |  |
| - 1. Have you watched pornography since you came to Burayu? | 1 Yes 2 No |  |
| - 1. Do you ever drink alcohol? IF YES. On how many days in the last month have you drunk alcohol? | Number of days Never |  |
| - 1. Do you ever chew khat? IF YES. On how many days in the last month have you chewed khat? | Number of days Never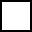 |  |
| - 1. Do you ever smoke cigarettes? IF YES. How many have you smoked in the last 7 days? | Number of Never  Cigarettes |  |

**Section 2: Sources of information on sexual and reproductive systems**

| 1. Have you ever received information related to **sexual and reproductive systems** since you moved to Burayu? | Yes  No | 2.4 |
| --- | --- | --- |
| 1. If Yes, From where? | 1. School 2. work place 3. family 4. relatives 5. Friends 6. health professionals 7. radio and television 8. Social media 9. Other (Specify… |  |
| 1. From whom or where, would you prefer to received (or prefer to have received) more information on this topic?   CIRCLE ONE ANSWER IN COL. 3 | 1. School 2. work place 3. family 4. relatives 5. Friends 6. health professionals 7. radio and television 8. Social media 9. Other (Specify… |  |
| - 1. Some schools have classes on puberty, on sexual and reproductive systems and on relationships between boys and girls. Did you ever attend school classes on any of these topics? |  | |

**Section 3: Current/most recent heterosexual relationship since migration**

| 3.1 Have you ever had a boyfriend? I mean someone to whom you were sexually or emotionally attracted and whom you 'dated' (Since you came to Burayu town) | Yes 1  No 2 | | Section 4 |
| --- | --- | --- | --- |
| 3.2 How old is that person? *Probe for current age* | Age | |  |
| 3.3 When you started your relationship, was your boyfriend was single, married, divorced or separated? | Single 1  Married 2  Divorce 3  Separated 4 | |  |
| 3.4 During the time you were/have been ‘dating’ your sexual partner did you 'date'/have you ‘dated’ anyone else? | Yes 1  No 2 | |  |
| Have you ever changed partner frequently? I mean over short period of time like with in less than 3 months | Yes 1  No 2 | |  |
| 3.5 How would you describe your relationship with your partner? Was (is) it (a) a casual friendship; (b) a serious relationship but with no intention of marriage; or (c) an important relationship that might lead to marriage? | (a) Casual 1   1. Serious 2 2. Important/might lead to marriage 3 3. Engaged to be married 4 | | 3.7 |
| 3.6 And how do you think NAME would describe her /his relationship to you? (a) as a casual friendship; (b) a serious relationship but with no intention of marriage; (c) an important relationship that might lead to marriage? | (a) Casual 1  (b) Serious 2  (c) Important/might lead to marriage  D engaged to be married | |  |
| 3.7 Did you and NAME have any physical contact, such as holding hands, hugging or kissing? | Yes 1  No 2 | |  |
| ***QUESTION 3.8 - 3.39 ARE ONLY FOR THOSE WHO HAVE EXPERIENCED PENETRATIVE SEX*** | | | |
| - 1. Now I have some question about the first time that you had sexual intercourse. How old were you at that time? | | AGE |  |
| - 1. Think back to the first time you had sex with NAME - I mean the first time that the penis was in the vagina. Would you say. READ OUT  1. I forced NAME to have intercourse against her/his will 2. I persuaded NAME to have intercourse 3. NAME persuaded me to have intercourse 4. NAME forced me to have intercourse 5. We were both equally willing | | (a) I forced 1  (b) I persuaded 2  (c) NAME persuaded 3  (d) NAME forced 4  (e) Both willing 5 |  |
| - 1. And would you say it was planned or unexpected? | | Planned 1  Unexpected 2 |  |
| - 1. How would describe your relationship to that person? PROBE | | 1. Boyfriend 2. Stranger/relative/other person but willingly 3. Stranger person but forced me 4. One night stand 5. Commercial sex partner |  |
| - 1. How old is that person? Probe for current age | | Age |  |
| - 1. What method did you use? | | 1. Condom 2. Pill 3. Injection 4. Post Pill 5. Withdrawal 6. IUD 7. Implant 8. Other……………… |  |
| - 1. Did you ever discuss contraception and STIs?with NAME? IF YES Did you discuss contraception and STIs before or after you first had intercourse? | | Before first intercourse 1  After first intercourse 2  Never 3 |  |
| - 1. Do you use condom during sexual intercourse? | | Yes 1  No 2 |  |
| - 1. Do you use it consistently on each intercourse? | | Yes 1  No 2 |  |
| - 1. Where did you get condom? | | 1. Shop 2. Pharmacy 3. Public Health care facility 4. Private Health care facility 5. From friends 6. Hotels 7. Others 8. I don’t know |  |
| - 1. Whose decision was to use condom? Was it mainly your decision, partner's decision or a joint decision? | | 1. My decision 2. NAME'S decision 3. Joint decision |  |
| - 1. Do you refuse to have sex if your partner refuses to use condom? | | 1. Yes 2. NO |  |
| - 1. Some young people are forced to have sexual intercourse against their will by a stranger, a relative or an older person. Has this ever happened to you? | | Yes 1  No 2 | 3.22 |
| - 1. How many different strangers, relatives or older persons have forced you to have sex against your will? | | No. |  |
| - 1. Some young people have 'one night stands' (*use local terms*), perhaps after a party or after drinking? Has this ever happened to you? | | Yes 1  No 2 | 3.26 |
| - 1. How many 'one night stands' have you had? | | No. |  |
| - 1. Did you or the sexual partner do anything to avoid a pregnancy / STIs on these occasions? IF YES Always or sometimes? | | 1. Always 2. Sometimes 3. Never |  |
| - 1. Some young people receive money or gifts in exchange for sexual intercourse. Has this ever happened to you? | | 1. Yes 2. No |  |
| - 1. Some young people do sexual intercourse in exchange for food or drink invitation. Has this ever happened to you since you came to burayu? | | 1. YES 2. NO |  |
| - 1. Did you or the sexual partner do anything to avoid a pregnancy / STIs on these occasions? IF YES Always or sometimes? | | 1) Always  2) Sometimes  3) Never |  |
| 3.40 Were you ever concerned that you might catch AIDS or another sexually transmitted disease from your sexual partner? IF YES Very or somewhat? | | Very concerned 1  Somewhat concerned 2  Not concerned 3 |  |

THIS SECTION IS ONLY FOR THOSE WHO HAVE NEVER EXPERIENCED SEXUAL INTERCOURSE

| People may have mixed reasons for not having intercourse. I will read out some reasons. Please tell me for each reason whether it applies to you or not. | Applies | Not applies | Don't Know/ Not Sure |  |
| --- | --- | --- | --- | --- |
| 4.1 I don't feel ready to have sex. | 1 | 2 | 3 |  |
| 4.2 I have not had the opportunity. | 1 | 2 | 3 |  |
| 4.3 I think that sex before marriage is wrong | 1 | 2 | 3 |  |
| 4.4 I am afraid of getting pregnant | 1 | 2 | 3 |  |
| 4.5 I am afraid of getting HIV/AIDS or another sexually transmitted infection.  I don’t know the reason | 1 | 2 | 3 |  |
| - 1. And now I have a question about your future plans about sexual intercourse. Which of these statement best describes your plans? READ OUT  1. I plan to wait until marriage 2. I plan to wait until I am engaged to be married 3. I plan to wait until I find someone I love 4. I plan to have sexual intercourse when an opportunity comes along | 1. Marriage 2. Engagement 3. Love 4. Opportunity | | |  |
| - 1. Do you feel any pressure from others to have sexual intercourse? IF YES A great deal or a little? | A great deal 1  A little 2  None 3 | | | Section 5 |
| - 1. From whom do you feel pressure? PROBE CIRCLE ALL THAT APPLY | Friends 1  Relatives 2  Work colleagues 3  Partner/special friend 4  Other ………………………………………5  …………………………………………………. | | |  |

**Section 5: Knowledge of HIV/AIDS and sexually transmitted diseases**

| 5.1 Have you heard of HIV or AIDS (*use local terms*)? | Yes 1  No 2 | | | | 5.5 |
| --- | --- | --- | --- | --- | --- |
| I am now going to read you some statements about HIV/AIDS. Please tell me whether you think the statement is true, or false, or whether you don't know. | True | | False | Don't know |  |
| 5.2 It is possible to cure AIDS | 1 | 2 | | 3 |  |
| 5.3 A person with HIV always looks emaciated or unhealthy in some way | 1 | 2 | | 3 |  |
| 5.4 People can take a simple test to find out whether they have HIV | 1 | 2 | | 3 |  |
| 5.5 Apart from HIV/AIDS, there are other diseases that men and women can catch by having sexual intercourse. Have you heard of any of these diseases? | Yes 1  No 2 | | | | SECTION 6 |
| - 1. What are the signs and symptoms of a sexually transmitted disease in a man? PROBE   CIRCLE EACH MENTIONED | Discharge from penis 1  Pain during urination 2  Ulcers/sores in genital area 3  Other………………………………  Other………………………………  D.K. any signs 8 | | | |  |
| - 1. And what are the signs or symptoms when a woman is infected? | Vaginal discharge 1  Pain during urination 2  Ulcers/sores in genital area 3  Other………………………………  Other………………………………  D.K. any signs 8 | | | |  |
| - 1. where could people obtain treatment needed for a sexually transmitted disease? PROBE Any other places?   CIRCLE EACH MENTIONED | 1. Shop 2. Pharmacy 3. hospital/health centre/clinic 4. Religious organization 5. Traditional healers 6. Other (SPECIFY)…………………… 5 | | | |  |

**Section 6: Condom knowledge and attitudes**

| CONDOMS KNOWN | CONDOMS NOT KNOWN | | | SECTION 7 |
| --- | --- | --- | --- | --- |
| - 1. SEE Q3.17 AND 3. 19 ON PAGE 6 &7   Respondent has experienced  sexual intercourse | Respondent has not  experienced sexual  intercourse | | | 6.5 |
| - 1. Have you or a partner ever used a condom? | Yes 1  No 2 | | | 6.6 |
| - 1. Have you ever experienced a condom that split or broke during intercourse? | Yes 1  No 2 | | | 6.6 |
| - 1. Have you ever seen a condom? | Yes 1  No 2 | | |  |
| - 1. People have different opinions about condoms. I will read out some opinions. For each one, I want you to tell me whether you agree or disagree, or whether you don't know | Agree | Don't know/not sure | Disagree |  |
| - 1. Condoms are an effective method of preventing pregnancy | 1 | 2 | 3 |  |
| - 1. Condoms can be used more than once | 1 | 2 | 3 |  |
| - 1. A girl can suggest to her boyfriend that he use a condom | 1 | 2 | 3 |  |
| - 1. Condoms are an effective way of protecting against HIV/AIDS | 1 | 2 | 3 |  |
| - 1. Condoms are suitable for casual relationships | 1 | 2 | 3 |  |
| - 1. Condoms are suitable for steady, loving relationships | 1 | 2 | 3 |  |
| - 1. It would be too embarrassing for someone like me to buy or obtain condoms | 1 | 2 | 3 |  |
| - 1. If a girl suggested using condoms to her partner, it would mean that she didn't trust him | 1 | 2 | 3 |  |
| - 1. Condoms reduce sexual pleasure | 1 | 2 | 3 |  |
| - 1. If unmarried couples want to have sexual intercourse before marriage, they should use condoms | 1 | 2 | 3 |  |

**Section 7: Sexuality, gender and norms**

| Young people have various views about relationships. I will read you out some views. For each one, please tell me whether you agree or disagree? |  |  |
| --- | --- | --- |
| 7.1. I believe it's all right for unmarried boys and girls to have dates (USE LOCAL TERM) | Agree 1  Don’t know/not sure 2  Disagree 3 |  |
| 7.2. I believe it's all right for boys and girls to kiss hug and touch each other. | Agree 1  Don’t know/not sure 2  Disagree 3 |  |
| 7.3 I believe there is nothing wrong with unmarried boys and girls having sexual intercourse if they love each other. | Agree 1  Don’t know/not sure 2  Disagree 3 |  |
| 7.4 I think that sometimes a boy has to force a girl to have sex if he loves her. | Agree 1  Don’t know/not sure 2  Disagree 3 |  |
| 7.5 A boy will not respect a girl who agrees to have sex with him. | Agree 1  Don’t know/not sure 2  Disagree 3 |  |
| 7.6 Most girls who have sex before marriage regret it afterwards. | Agree 1  Don’t know/not sure 2  Disagree 3 |  |
| 7.7 A boy and a girl should have sex before they become engaged (USE LOCAL TERM) to see whether they are suited to each other. | Agree 1  Don’t know/not sure 2  Disagree 3 |  |
| 7.8 I believe that girls should remain virgins until they marry. | Agree 1  Don’t know/not sure 2  Disagree 3 |  |
| 7.9 Most of my friends think that one-night stands are OK. | Agree 1  Don’t know/not sure 2  Disagree 3 |  |
| 7.10 It's all right for boys and girls to have sex with each other provided that they use methods to stop pregnancy. | Agree 1  Don’t know/not sure 2  Disagree 3 |  |
| 7.11 Most of my friends who have sex with someone use condoms regularly. | Agree 1  Don’t know/not sure 2  Disagree 3 |  |
| 7.12 I am confident that I can insist on condom use every time I have sex. | Agree 1  Don’t know/not sure 2  Disagree 3 |  |
| 7.13 I would never contemplate having an abortion myself or for my partner. | Agree 1  Don’t know/not sure 2  Disagree 3 |  |
| 7.14 It is mainly the woman's responsibility to ensure that contraception is used regularly. | Agree 1  Don’t know/not sure 2  Disagree 3 |  |
| 7.15 I think that you should be in love with someone before having sex with them. | Agree 1  Don’t know/not sure 2  Disagree 3 |  |
| 7.16 I feel that I know how to use a condom properly. | Agree 1  Don’t know/not sure 2  Disagree 3 |  |
| 7.18 I would refuse to have sex with someone who is not prepared to use a condom. | Agree 1  Don’t know/not sure 2  Disagree 3 |  |

**Section 8 Question on social Use of Social media and pornography**

| - 1. Do you use social media? | 1. Yes 2. No |  |
| --- | --- | --- |
| 8.2 If your answer is yes for Q#801; then, which social media have you used? | 1. Facebook 2. Viber 3. WhatsApp 4. You tube 5. Instagram 6. Other (specify)…………. |  |
| 8.3 How often do you use it? | 1. Many times a day 2. Everyday 3. Every week 4. Once I a while 5. Other (specify)…………………. |  |
| 8.4  For what purpose do you use it? | 1. To interact with my friends 2. For entertainment 3. To watch NEWS updates 4. To watch movies 5. To watch pornography 6. Other (specify)…………. |  |
| 8.5 Have you ever tagged to sexual arousing picture or video in social media? | 1. Yes 2. No |  |
| 8.6 If your answer is yes for Q number 505, then what was your feeling? | 1. It was normal / I felt nothing strange 2. It arouse me for sex 3. It made me think to have boyfriend sooner than my plan. 4. It made me to start sex early 5. Other (specify)……………………. |  |
| 8.7 Have you viewed pornographic materials (sex film or photographs) in the last 3 months? | 1. Yes 2. No |  |
| 8.8 What happened after that? | 1. Nothing has happened 2. I had sex with my boy/ girl friend 3. I decided to have boy/girl friend 4. I had casual sex 5. Other (specify)…………….. |  |
| 8.9 How old were you when you viewed pornographic materials for the first time? | ………………years old |  |

**~END~**
